# D'oh! Genes and Environment Cause Crohn's Disease

**DOI:** 10.1016/j.cell.2010.06.015

**Published:** 2010-06-25

**Authors:** John A. Todd

**Affiliations:** 1 Juvenile Diabetes Research Foundation/Wellcome Trust Diabetes and Inflammation Laboratory, Department of Medical Genetics, Cambridge Institute for Medical Research, University of Cambridge, Cambridge CB2 0XY, UK

## Abstract

Information obtained from genome-wide association studies has cracked open the biology of common chronic diseases by identifying genes that predispose individuals to these disorders. Cadwell et al. (2010) now demonstrate that a viral infection, a toxic insult to the gut, commensal bacteria, and a Crohn's disease susceptibility gene collude to cause inflammatory disease in the mouse gut.

## Main Text

A revolution is occurring in our understanding of the genetic basis of common chronic diseases such as arthritis, Crohn's disease, and cancer. Genome-wide association studies (GWAS) are identifying a plethora of common sequence variants of genes and of putative regulatory regions in or near genes that predispose an individual to developing a particular disease. This flood of new information is made possible by dramatic improvements in the genotyping of DNA polymorphisms across the human genome, increased levels of statistical rigor during the interpretation of results, and the large-scale collection of clinical resources. Nevertheless, doubts concerning the value of GWAS have arisen because in many cases a particular polymorphism only slightly increases an individual's risk for developing that disorder. Hence, some researchers ask, how can such a small genetic effect provide useful insights into the mechanisms of the disease? However, more pertinent questions are, which particular genes mapped by GWAS are involved in the etiology or cause of the disease and how do these genes act?

**Figure 1:**
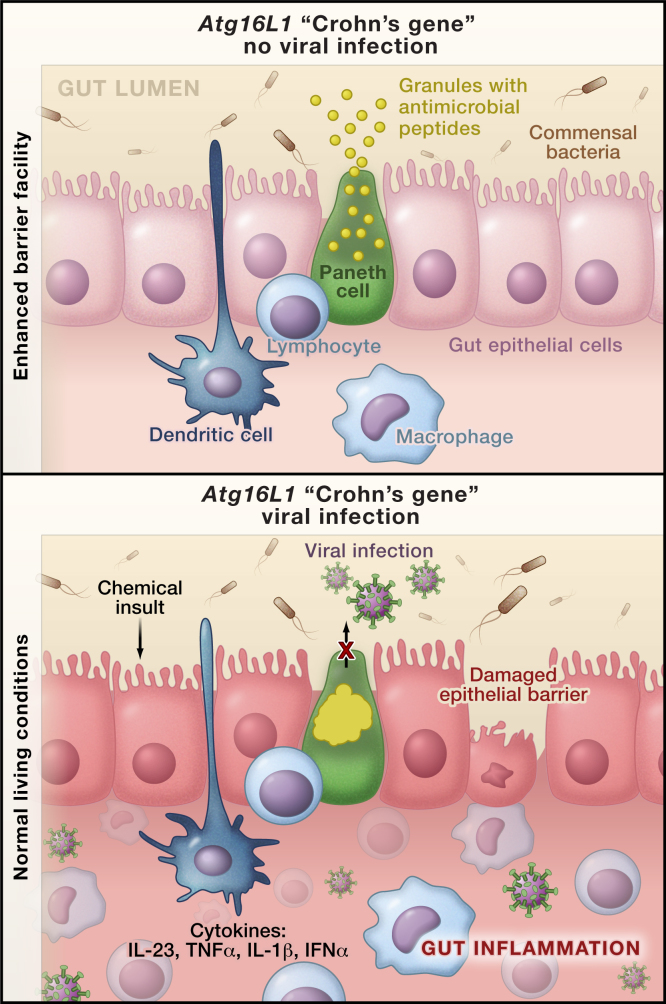
Environmental and Genetic Factors Required for Crohn's-like Disease in Mice An allele of the human autophagy gene *ATG16L1* predisposes individuals to an inflammatory bowel disorder, called Crohn's disease. Patients homozygous for this disease allele display morphological abnormalities in the granules of their specialized gut epithelial cells, called Paneth cells (Cadwell et al., 2008). (Top) Cadwell et al. (2010) now show that in a mouse model of Crohn's disease in which the mouse *Atg16L1* ortholog is disrupted, the Paneth cells have a normal phenotype when the mice are raised in virus-free enhanced barrier facilities. (Bottom) However, in conventional living conditions these mutant mice are infected with norovirus and develop similar abnormalities in the Paneth cells as those observed in patients with Crohn's disease. This pathology also depends on the interactions of commensal bacteria, the gut innate immune system, and the production of proinflammatory cytokines.

One common disorder for which GWAS have been spectacularly successful is a severe form of gut inflammation called Crohn's disease. GWAS of Crohn's disease patients provided the first clues that alterations in the expression and activities of genes known to function in autophagy (i.e., the pathway that degrades intracellular components, including pathogens) may predispose individuals to inflammation of the small intestine (Wellcome Trust Case Control Consortium, 2007). Two earlier studies, one by Cadwell et al. (2008) and another by Saitoh et al. (2008), took the next steps after the GWAS investigations by modeling in mice the effects of the Crohn's disease gene *ATG16L1*. This gene was already associated with autophagy, and in both studies, mice engineered with deficiencies in the *Atg16L1* gene displayed gut inflammatory phenotypes not previously associated with autophagy. Reducing the function of Atg16L1 in mice caused significant changes to specialized gut epithelial cells, called Paneth cells. These cells contain granules packed with antimicrobial peptides (Figure 1, top). Disrupting the mouse *Atg16L1* ortholog decreased how much antimicrobial peptides their Paneth cells produced (Cadwell et al., 2010), and it increased the secretion of the proinflammatory cytokines IL-1β and IL-18 by macrophages (Saitoh et al., 2008). Now, in this issue of *Cell*, Cadwell et al. (2010) characterize environmental factors that interact with reduced functionality of ATG16L1 to cause inflammation in the small intestines of mice. Common diseases are the result of obligatory interactions between many genetic susceptibility alleles and equally numerous, but largely unknown, environmental factors (Todd, 2010). These new results by Cadwell et al. (2010) demonstrate how findings from GWAS may help to provide the first glimpses of the new frontier of gene-environment interactions.

In a serendipitous observation, Cadwell et al. (2010) found that moving the *Atg16L1-*deficient mice from conventional barrier living conditions to an enhanced barrier facility (i.e., a “super pathogen-free” environment) surprisingly protected the mice from the Paneth cell phenotype of aberrant granule packaging (Figure 1). It could have taken years to pinpoint the key difference between the two living conditions, yet the authors, with foresight and expertise in virology, did just this remarkably quickly. They found that the mice living a conventional lifestyle were infected with norovirus, which induced the Paneth cell abnormalities and gut inflammation when the *Atg16L1* mutation was present. Furthermore, the effect was specific for a particular strain of norovirus (MNV CR6) and required persistent infection in the *Atg16L1-*deficient mice (Cadwell et al., 2010).

In accord with Saitoh et al. (2008), Cadwell and colleagues (2010) also showed that exposure of the *Atg16L1-*deficient mice to a toxin induces intestinal inflammation that resembles Crohn's disease. Further, the severity of this environmental insult depends not only on disruption of the *Atg16L1* gene and infection with norovirus but also on the presence of commensal bacteria in the gut (i.e., the microbiome). Finally, they reported that the proinflammatory cytokines IFNγ and TNFα were required for the pathology. Thus, in a mouse model, the authors connect all of the factors one would expect to be involved in the origin of a common immune disease: a time-dependent and complex interaction among common gene polymorphisms, the immune system, the microbiome, environmental toxins, and the individual's infection history (Cadwell et al., 2010).

A link between the activity of the gut microbiome and the development of a chronic disease is not without precedent. Previous studies have implicated this highly diverse bacterial soup in obesity and type 1 diabetes (Wen et al., 2008). In addition, some investigators have puzzled over the observation that the common genetic variants identified by GWAS explain little of the familial clustering of these diseases. Because babies inherit their microbiome from their mothers during birth, microbiome transmission could be a major factor in explaining the apparent strong heritability of many common diseases (Todd, 2010). Characterizing how the microbiome contributes to health and disease is well underway, particularly with the recent advent of high-throughput “next-generation” DNA sequencing methods. Deep sequencing, which can detect sequences or mutations occurring at very low frequencies, is also being applied to the human virome, but this work is in its infancy (Virgin et al., 2009).

To be clear, Cadwell and coworkers are not arguing that Crohn's disease is caused by infection with norovirus or by any other single microbe. The environmental factors that predispose to and protect from Crohn's disease remain uncertain, but the balance among commensal and pathogenic gut bacteria and viral infections is likely to be part of the story. Cadwell et al. (2010) also do not reveal which cells in the mouse gut or its immune system are infected with murine norovirus. An intracellular receptor, interferon-induced helicase C domain 1 (IFIH1) or MDA5, is required to suppress murine norovirus infection (Virgin et al., 2009). However, although this receptor is widely expressed, the murine norovirus preferentially infects dendritic cells and macrophages of the immune system (McCartney et al., 2008).

Interestingly, mutant alleles of the *IFIH1* gene encoding this virus receptor are associated with protection from autoimmune diseases such as type 1 diabetes and systemic lupus erythematosus (Todd, 2010) (http://www.t1dbase.org/page/Regions/). Many studies have reported or proposed that infection with certain viruses and an increase in production of the inflammatory molecule type 1 interferon are causes of these disorders (Todd, 2010). Moreover, the Toll-like receptors TLR7 and TLR8 expressed in the gut are also sensors of viral infections, and variations in their genes have been associated with type 1 diabetes and celiac disease (an inflammatory disorder of the small intestine) (Todd, 2010). In addition, Saitoh et al. (2008) reported that TLR7 is involved in the Atg16L1-mediated production of IL-1β. Combined with the IFIH1 findings, the new study by Cadwell and coworkers (2010) suggests that a variety of viruses may be cofactors in the development of immune diseases (Todd, 2010).

Despite the current revolution in human genetics and genomics, identification of genes that control susceptibility and resistance to infectious diseases still lags behind the explosion of information about gene variations implicated in chronic diseases. Researchers are still identifying the molecules that govern the persistence of viral infections, such as interleukin-10 and cytotoxic T-lymphocyte antigen 4 (CTLA-4) (Virgin et al., 2009). Variants of the genes encoding these molecules occupy central positions in the newly discovered genetic architecture of chronic immune diseases (Todd, 2010). Together with the new work of Cadwell et al. (2010), these studies make an urgent and compelling case for characterizing the human virome and defining its effects on physiology and gene expression. In addition, we need to understand how the virome interacts with polymorphisms in the host genome and how numerous toxins in the environment alter this complex interplay (Patel et al., 2010).

GWAS and genome-wide expression studies, coupled to gene-to-phenotype approaches and mouse disease models, not only will facilitate the identification of key environmental factors contributing to common disorders, but they also underpin the hope for more etiology-based development of new therapeutics. For example, data from the Crohn's disease GWAS are already yielding potential clinical insights. The immunosuppressant rapamycin, which induces autophagy in cultured cells, improved symptoms in a patient with refractory Crohn's disease (Massey et al., 2008).
